# Advances in Personalized Medicines along with Functional Genomics and Pharmacogenomics

**DOI:** 10.3390/jpm11100941

**Published:** 2021-09-22

**Authors:** Su-Jun Lee

**Affiliations:** Department of Pharmacology and Pharmacogenomics Research Center, College of Medicine, Inje University, Busan 50834, Korea; 2sujun@inje.ac.kr

State-of-the-art research on the human genome has produced remarkable research achievements in pharmacogenomics and functional genomics, and these research results are making an invaluable contribution to the advancement of personalized medicine. Advances in human genome research are being made from highly integrated next generation sequencing technologies, molecular biology techniques, cell and tissue fusion technologies, computational approaches, and various clinical studies. In addition, continuous technology development and the application of various omics are increasing basic data and potential for the realization of customized drug therapy. Although tremendous progress has been made in pharmacogenomics through human genetic research over the past 20 years, there are still many problems that remain to be solved, such as optimization of individual drugs and drug doses, comprehensive discovery of genetic factors affecting drug responses, and real-time monitoring of drug targets.

This Special Issue, Functional Genomics and Pharmacogenomics in Human Disease, covers 15 publications conducted by various groups, including nine original research papers, five review papers, and one case report. Original research papers consist of genetic discovery and functional characterization of *CYP1A2* and *CYP2C9* [1,2], genetic studies of oral cancer [3,4,5], chronic obstruction pulmonary disease (COPD) [6], asthma [7], diabetic kidney disease [8], and web-based pharmacogenomics tool development [9]. Review papers include the thiopurine treatments with *NUDT15* polymorphisms [10], lipid lowering therapies in familial hypercholesterolemia [11], incorporation of genetic addiction risk severity (GARS) to overcome the addiction pandemic [12], and the latest update on genetic variants of *UGT* [13] and *SULT* genes [14]. The case report includes pharmacogenomics of allopurinol and sulfamethoxazole/Trimethoprim [15].

The purpose of studying the relationship between genotype and phenotype is to provide the correct drugs and correct drug doses suitable for a phenotype and also to predict the progression of phenotype. Here, the phenotype includes drug responses, disease progressions, and diverse biosignals. However, it is very difficult to select a set of genetic variants or to decide on how many genetic variants are involved in the phenotype. There are various reasons why it is difficult to select a set of genetic variants. One of the reasons is that the number of genetic variants for which its biological functions have been identified is very limited compared to the huge number of discovered variants in humans. Information on changes in biological function of genetic variants provides scientific persuasiveness for clinical application of genetic analysis to both physicians and patients in the clinical setting, rather than relying on statistical significance and model prediction in case/control studies. Therefore, it is firmly believed that functional studies on rare variants should be actively and continuously performed.

Functional studies of the protein variants discovered in the *CYP1A2* [1] and *CYP2C9* [2] genes were conducted, and the studies identified multiple genetic variants with significantly reduced enzyme activity. This is expected to make a great contribution to drug metabolism, environmental substance metabolism, and toxicology research with regard to the CYP1A1 and CYP2C9 substrates. Oral cancer research was conducted with genetics and presented the risk of cancer occurrence, the sensitivity of cancer development, and the degree of cancer progression related to genetic variants. *MACC1* [3], *LINC00673* [4], and *IncRNA GASS5* [5] were investigated, and the correlation between the mutants of these genes and oral cancer was described. If additional genetic variants related to oral cancer are further studied on the present patients, it is expected to provide important information that is very practical for personalized treatment and prevention of oral cancer. Zihlif et al. [7] reported the significant relationship between the *ADAM33* genetic variant and the IgE level related to allergic reactions in 267 atopic patients, which provides substantial information related to the care of asthmatic patients. Due to the radical development of human genome sequencing technology, a huge number of genetic variants are being discovered, and the number of genetic variants related to pharmacogenomics is also being reported very vastly. Therefore, it is not access and obtain drug–genome interaction information from complex and many genetic variants, but John et al. [9] have developed a web-based pharmacogenomics tool (PharmaKU), which is expected to provide useful information to clinicians. Chronic obstructive pulmonary disease (COPD) is one of the diseases with high mortality worldwide, and it is known that genetic and environmental factors influence the disease. Interestingly, vitamin D levels are low in COPD patients and are known to increase the risk of morbid condition. El Shamieh et al. [6] investigated the correlation between the genotype of top 10 genes related to COPD and vitamin D level, and they reported that a mutant of the *FAM13A* gene (rs6837671) was associated with vitamin D level and increased the risk in COPD. Diabetic kidney disease (DKD) occurs mainly in diabetic mellitus patients and is the most common form of end-stage renal disease (ERSD). A microarray experiment was conducted and found that the ubiquitin protein ligase E3C (UBE3C) is a candidate gene that affects kidney function [8]. Then, it was reported that the *UBE3C* haplotype with the identified variants was correlated with chronic kidney disease. The results of this study are expected to provide data that will serve as a foundation for presenting a good predictive model by combining other related genetic variants and clinical factors.

In this Special Issue, several excellent review articles were introduced. First of all, Hindi et al. [11] pointed out that there are currently numerous genetic variants and many unknown variants scattered in a complex manner, making it difficult to classify them functionally and clinically. Therefore, it was suggested that collecting samples for familial-based clustered disease could be a good method for solving the complexity. As an example, they introduced familial hypercholesterolemia and pharmacogenomics in a review article [11]. Thiopurine S-methyltransferase (TPMT) is an enzyme involved in the metabolism of thiopurine drugs, and its genotype is used as a good genetic marker to prevent and predict the side effects of these drugs in Caucasians. However, since the frequency of *TPMT* genetic variants used as predictive markers for adverse drug reactions is quite low in Asians, its application in Asians is very limited. Yoshiro Saito’s group [10] described the recent update of nudix hydrolase 15 (NUDT15) genetic information with thiopurine-induced side effects and its application in Asian races. UGT enzyme is a representative phase II enzyme group and plays an important role in the metabolism of fatty acids, hormones, and drugs. The latest update of genetic variants of UGTs and disease-related information were described [13]. SULT1E1 plays an important role in hormone metabolism and the metabolism of certain drugs, but there has been lack of systematic classification of genetic information of this enzyme with regard to human diseases. Yi et al. [14] have summarized and updated the latest information on expression regulation, clinical role, and genetic variation of this enzyme. Blum et al. [12] proposed a method of reducing the risk of opioid use disorder by evaluating reward-related genetic variants and risk-related genetic variants by applying genetic an addiction risk severity (GARS) test into the dopamine homeostasis-related clinical settings. Most dermatologists take action quickly when severe cutaneous adverse drug reactions (SCAR) occur, but the efforts to prevent them are relatively weak. Ikediobi et al. [15] described how dermatologists used pharmacogenomic information to prevent and diagnose SCAR induced by allopurinol and sulfamethoxazole/trimethoprim, using literature review and clinical cases as examples. This case report is judged to provide useful information with high utility in actual clinical practice.

Knowledge of pharmacogenomics is becoming important in almost all field of medicine, since a growing number of patients report beneficial effects by genotype analysis. Cutting-edge research in pharmacogenomics aims integrate pharmacogenomics into the clinical field. For pharmacogenomics to be used more extensively in clinical practice, a greater amount of genomic data with functional evidence should be accompanied. The aim of the present Special Issue is to provide functional explanations for individual variations in drug toxicities, drug resistances, and drug sensitivities. Although we did not cover pharmacogenomics in more diverse fields, all authors of this Special Issue are proud to provide substantial and specific pharmacogenomic information for the realization of personalized medicine in their respective fields. We hope that the results, research methods, research theories, and information provided in this Special Issue will be of great help in achieving the personalized medicine we all want.
